# Measles—an ENT diagnosis?

**DOI:** 10.1002/ccr3.8160

**Published:** 2023-11-04

**Authors:** Tiago Lourenço Coelho, Nuno Dias Silva, Hugo Figueiredo, Ricardo Caiado

**Affiliations:** ^1^ Department of Otorhinolaryngology Centro Hospitalar e Universitário de Coimbra Coimbra Portugal

**Keywords:** Koplik's spots, measles

## Abstract

Ear, Nose and Throat (ENT) should remain vigilant about the emergence of measles in non‐endemic countries. Clinical suspicion is crucial in identifying this disease, with Koplik's spots being a pathognomonic sign. Forming part of the differential diagnosis and helping to prevent potential outbreaks.

## Graphical abstract

Classic Koplik's spots in the oral cavity, a crucial indicator of measles infection.

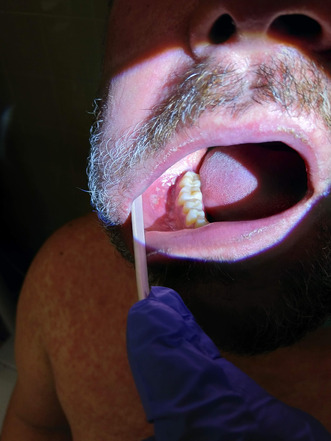

Measles is a severe viral infection characterized by fever, cough, runny nose, conjunctivitis, and a distinctive rash. It spreads through respiratory droplets and can lead to complications like pneumonia and encephalitis.^1^, ^2^, ^3^ Despite available effective vaccines, sporadic outbreaks can still occur due to a combination of global travel and inconsistent vaccination coverage.^2^

We report a case of a 42‐year‐old Caucasian male with a recent travel history to Brazil. He presented with a 3‐day history of fever, cough, odynophagia, and a progressive skin rash, appearing 10 days post‐return. His immunization records indicated an absence of documented measles vaccination. On admission, the tympanic temperature was 39.2°C. Physical examination revealed a cephalocaudal maculopapular rash that spread across his face, chest, and extremities (Figures 1 and 2), and multiple 1–2 mm gray–white spots on the buccal mucosa consistent with Koplik's spots (Figure 3).

**FIGURE 1:**
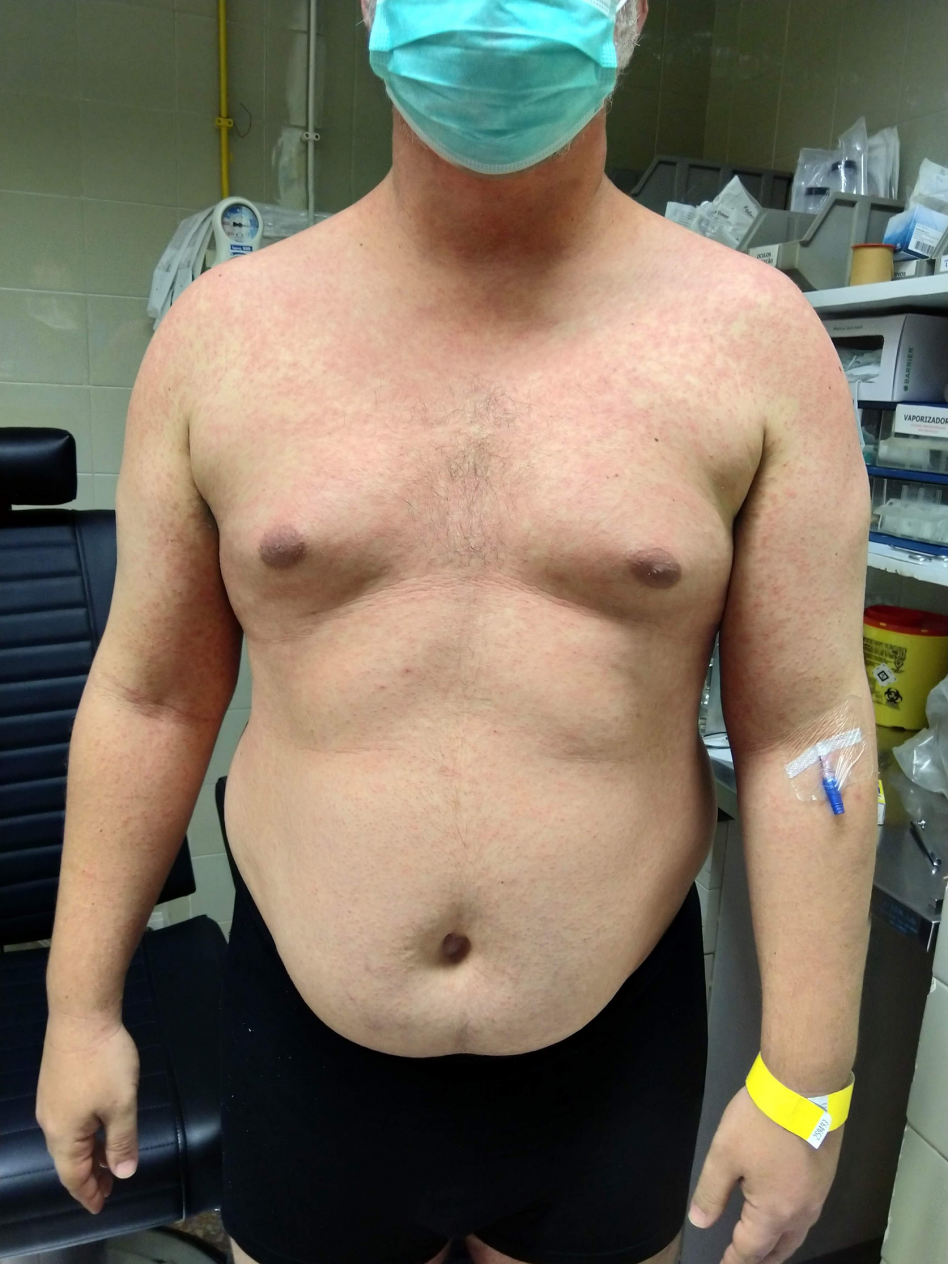
Cephalocaudal maculopapular rash in the chest and extremities.

**FIGURE 2:**
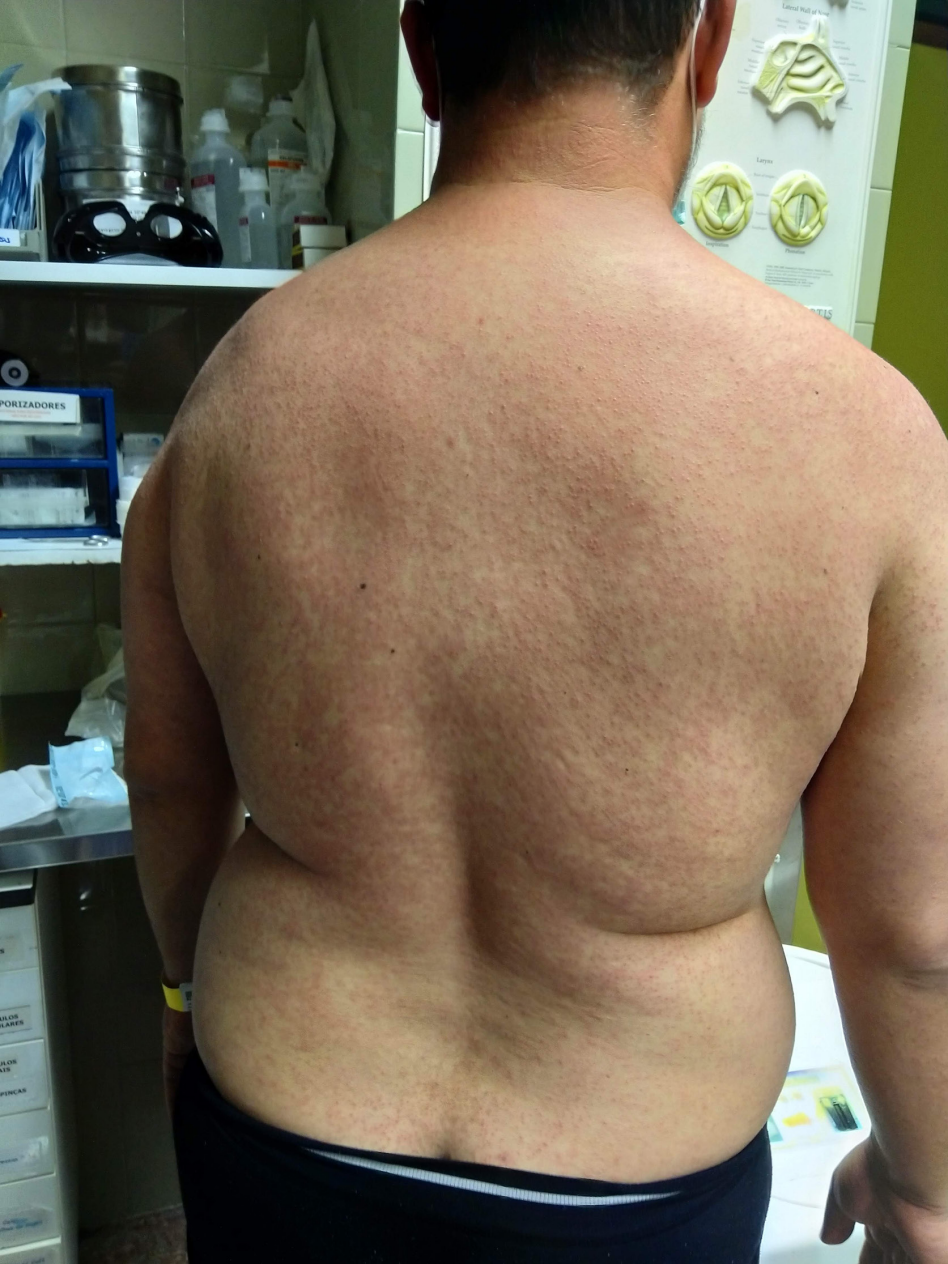
Cephalocaudal maculopapular rash in the dorsal region.

**FIGURE 3:**
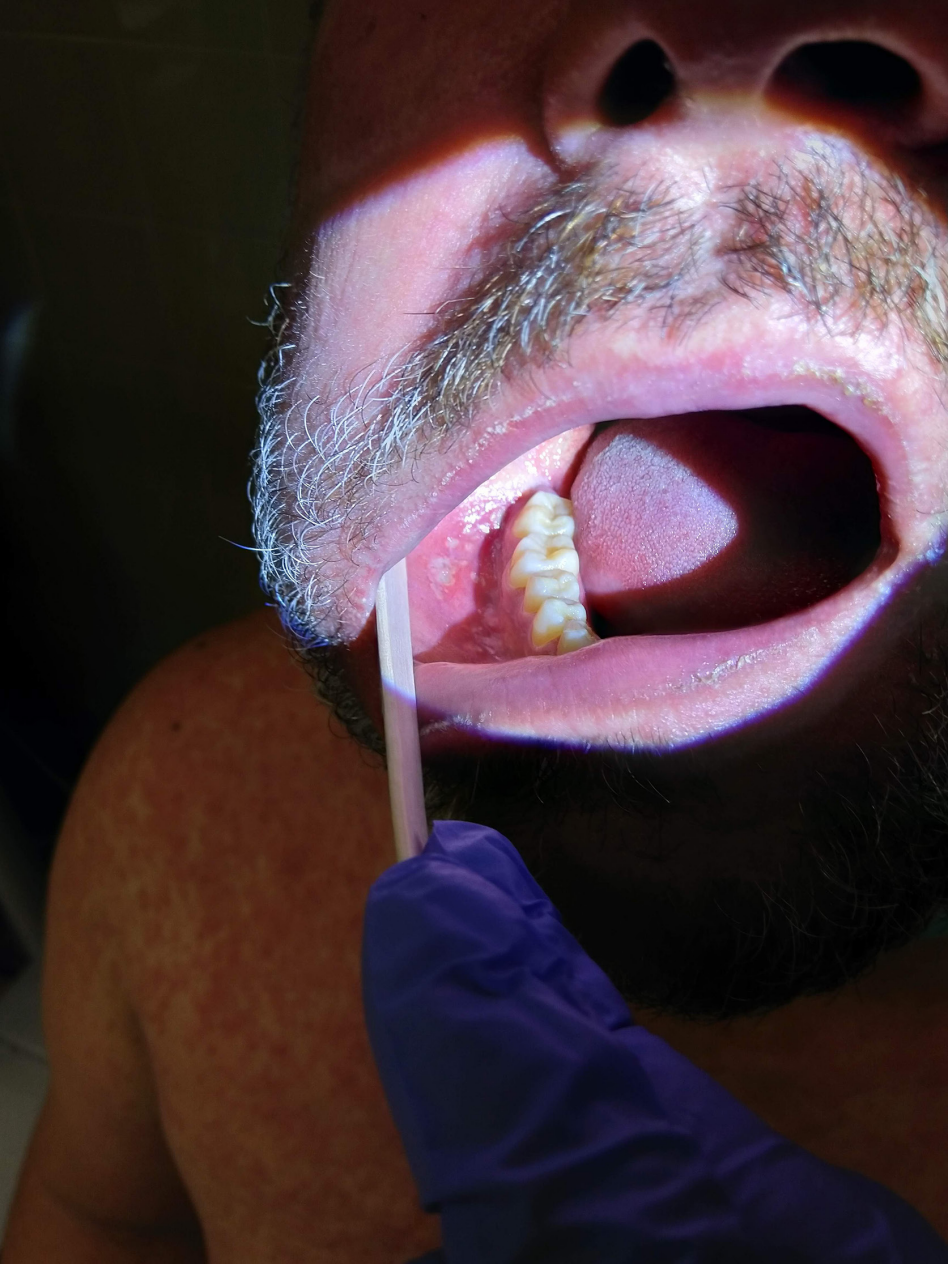
Koplik's spots on the buccal mucosa.

Due to the combination of clinical symptoms, a recent travel history to an area with known measles outbreaks, and a confirmed absence of immunization against the virus, measles emerged as the primary diagnosis.

Following the suspected diagnosis, rigorous infection control measures were promptly implemented. The patient was isolated in a specialized room equipped with negative pressure ventilation. All healthcare staff attending to the patient wore N95 respirators and strictly followed rigorous hand sanitizing protocols. Additionally, the immunization status of healthcare workers was verified, and those who were not immune were prevented from having direct contact with the patient.

Oropharyngeal swab, urine, and blood specimens were taken. The diagnosis was confirmed by positive polymerase chain reaction (PCR) for measles. Serological tests using enzyme immunoassay were also performed revealing IgM antibodies against measles (IgM titer 8.4, positive ≥1.1; IgG titer 9.0; positive ≥16.5), compatible with acute infection.

He received supportive care and within a few weeks all his symptoms disappeared.

In Portugal, measles is a notifiable disease, which means that healthcare professionals are required to promptly report any suspected or confirmed cases to health authorities. This rapid notification allows for the implementation of public health measures, including epidemiological investigation, patient isolation, vaccination of individuals at risk, and public awareness campaigns. In this particular case, following these standard protocols, there were no reported instances of transmission, underscoring the effectiveness of timely interventions.

There is an increase in new cases of measles in developed countries where vaccination is optional. This case demonstrates the importance of continuing to include measles in the differential diagnosis, even in countries where this disease has been eliminated, since cases can arise in other countries and easily cause outbreaks.

Detailed patient histories and thorough physical examinations are essential in identifying suspected cases.

The role of the otorhinolaryngologist as a public health agent is reinforced preventing transmission and implement control measures.

Early isolation and adherence to infection control protocols are paramount in preventing healthcare‐associated spread. Attention should also be drawn to the need for measles vaccination for all health care providers.

## AUTHOR CONTRIBUTIONS

**Tiago Lourenço Coelho:** Conceptualization; data curation; formal analysis; investigation; methodology; project administration; writing – original draft; writing – review and editing. **Nuno Dias Silva:** Data curation; investigation; supervision; writing – review and editing. **Hugo Figueiredo:** Data curation; writing – review and editing. **Ricardo Caiado:** Supervision; validation; writing – review and editing.

## FUNDING INFORMATION

The authors declare that there are no sources of financing.

## CONFLICT OF INTEREST STATEMENT

The authors declare that they have no conflicts of interest related to this work.

## ETHICS STATEMENT

The authors declare that the procedures were followed according to the regulations established by the 2013 Helsinki Declaration of the World Medical Association. The authors declare having followed the protocols in use at their working center regarding patients' data publication.

## CONSENT

Written informed consent was obtained from the patient to publish this report in accordance with the journal's patient consent policy.

## Data Availability

Data sharing not applicable—no new data generated.
